# Phytochemical, Therapeutic, and Ethnopharmacological Overview for a Traditionally Important Herb: *Boerhavia diffusa* Linn.

**DOI:** 10.1155/2014/808302

**Published:** 2014-05-14

**Authors:** Shikha Mishra, Vidhu Aeri, Praveen Kumar Gaur, Sanjay M. Jachak

**Affiliations:** ^1^Department of Pharmacognosy and Phytochemistry, Faculty of Pharmacy, Jamia Hamdard, Hamdard Nagar, New Delhi 110062, India; ^2^Department of Pharmaceutics, ITS Pharmacy College, Muradnagar, Ghaziabad 201006, India; ^3^Department of Natural Products, National Institute of Pharmaceutical Education & Research, S.A.S. Nagar, Punjab 160062, India

## Abstract

*Boerhavia diffusa* (BD) is a plant of *rasayana* category as per ayurvedic claims. It is reported to possess antiaging, disease prevention, and life strengthening activities which hold enormous influence in disease burden and affordability/availability of healthcare in the world. *Objective.* This paper has been compiled to comment on the studies reported for BD to highlight its chemical and therapeutic potential along with its ethnopharmacological considerations. *Methods.* In the present paper, a detailed account of chemical constituents and pharmacological activities has been presented. All the findings were correlated with modern pharmacological activities to appraise the value of BD. * Results.* Chemical analysis of BD gives a wide variety of chemical constituents, namely, rotenoids, flavonoids, xanthones, purine nucleoside, lignans, and steroids. Various ethnopharmacological reports emphasize its role in disorders of reproductive system, gastrointestinal system, respiratory system, urinary system, hepatic system/jaundice, cardiovascular system, and cancer. *Conclusions.* The studies on the therapeutic activities of BD range from studies on crude extracts to isolated compounds; however some of the studies require sophistication and validated results. BD is a plant of enormous importance in the purview of its chemical and therapeutic properties.

## 1. Introduction

*Boerhavia diffusa* (BD) Linn. (Nyctaginaceae) is a well-known medicinal plant in traditional Indian medicine as well as other parts of world, for example, Southern American and African continent. Its various parts and especially roots have been used for gastrointestinal, hepatoprotective, and gynecological indications in above mentioned parts of the world and also throughout India. In ayurvedic texts, more than 35 formulations of different types contain it as major ingredient.

In Ayurveda, BD has been classified as “rasayana” herb which is said to possess properties like antiaging, reestablishing youth, strengthening life and brain power, and disease prevention, all of which imply that they increase the resistance of the body against any onslaught, in other words, providing hepatoprotection and immunomodulation [1].

BD has been widely studied for its chemical constituents and therapeutic activities. The roots are the source of a novel class of isoflavonoids known as rotenoids, flavonoids, flavonoid glycosides, xanthones, purine nucleoside, lignans, ecdysteroids, and steroids. Various animal studies and trials have confirmed the presence of activities, for example, immunomodulation, hepatoprotection, antifibrinolysis, anticancer activity, antidiabetic activity, anti-inflammation, and diuresis. In this paper, traditional uses, chemical constituents, and reported pharmacological activities have been summarized to present the chemical and therapeutic potential of this plant. Present review also provides an ethnopharmacological appraisal of an important medicinal herb.

**Figure 1:**
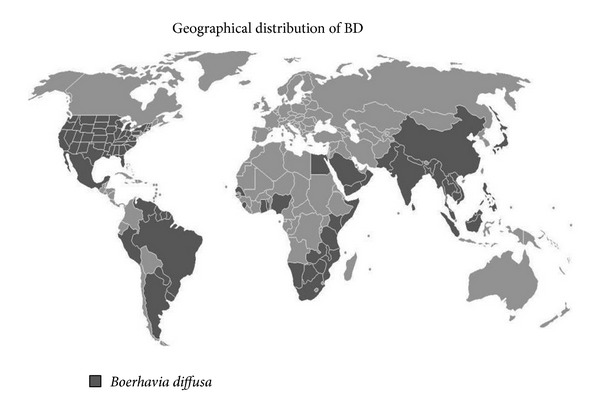
Worldwide distribution of BD.

*Botany and Substitutes. Boerhavia* genus is a collection of 40 tropical and subtropical species. It is found as a weed during rainy seasons in Indian, Northern and Southern American continents and South Eastern Africa. Boerhavia was named after Hermann Boerhaave, a famous Dutch physician of the 18th century, while the species got the name from its typical diffuse branching. Two views have been taken on the application of the name BD: a broad view regarding several* Boerhavia* taxa (including* Boerhavia repens* L. and* Boerhavia coccinea* Mill.) as a single very variable species and a restricted concept in which BD is applied to the taxon with an apparently terminal panicle. It is called by several different names owing to its wide distribution throughout the world (Figure 1), that is, alena (Hawaii); erva tostão, agarra-pinto, and amarra-pinto (Brazil); hogweed (Barbados); red spiderling, spreading hogweed (English); Huang Xi Xin (Chinese); ipecacuanha de Cayenne (French Guiana); and hierba de cabra (Spanish).

In Indian context, BD goes by several names due to the variety of languages, that is, Assamese: Ranga Punarnabha; Bengali: Rakta Punarnava; Gujrati: Dholisaturdi, Motosatodo; Hindi: Gadapurna, Lalpunarnava; Kannada: Sanadika, Kommeberu, and Komma; Kashmiri: Vanjula Punarnava; Malayalam: Chuvanna Tazhutawa; Marathi: Ghetuli, Vasuchimuli, Satodimula, Punarnava, and Khaparkhuti; Oriya: Lalapuiruni, Nalipuruni; Punjabi: ltcit (Ial), Khattan; Tamil: Mukurattai (Shihappu); and Telugu: Atikamamidi, Erra galijeru.

However, one of its names in Sanskrit (kathilla, sophaghni, sothaghni, and varsabhu), varshabhu, has given rise to a long standing confusion on identity. As per modern taxonomy, varshabhu is the name for* Trianthema portulacastrum*. Both of these plants also bear morphological similarities.

## 2. Chemistry

BD is a good source of nutritional supplements as reported by Miralles and Ujowundu. Miralles et al. reported 15 amino acids (6 essential) in the whole plant and 14 amino acids (7 essential) in the roots along with isopalmitate acetate, behenic acid, arachidic acid (6.3%), and saturated fatty acids (38%) [2]. Ujowundu et al. [3] accounted the presence of vitamins C, B_3_, and B_2_ (44.80, 97.00 mg, and 22.00 mg) along with calcium (174.09 mg) in roots. In various tribal areas, BD roots as well as whole plant have been reported to be used as culinary ingredient. Based on the above studies, this use can be a validated claim.

**Figure 2:**
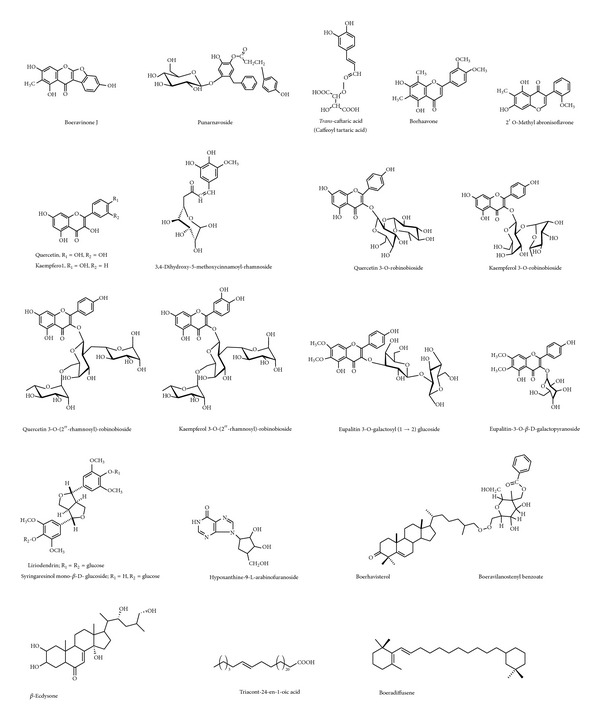
Major chemical constituents isolated from BD.

**Figure 3:**
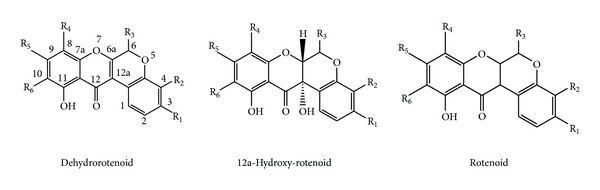
Categories of rotenoids.

**Figure 4:**
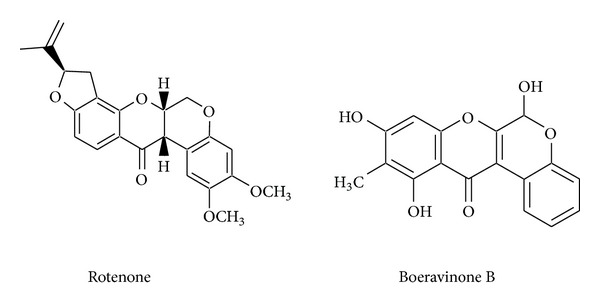
Prototype compound of rotenoid category and chemical marker of BD.

BD contains various categories of secondary metabolites, for example, flavonoid glycosides, isoflavonoids (rotenoids), steroids (ecdysteroid), alkaloids, and phenolic and lignan glycosides.  Table 1 gives an account of various chemical constituents isolated till date from BD along with activity observed for that compound.  Figure 2 shows the structural diversity of the compounds isolated from BD.  Figure 3 and Table 2 give a detailed survey of compounds from isoflavonoids category. Recently a rapid method was developed for quantitative estimation of boeravinones in BD [4]. Rotenoids are isoflavonoids derivatives, with a prototype compound named rotenone, which is a mitochondrial inhibitor (Figure 4). It causes inhibition of mitochondrial electron transport chain at complex I [5]; however the “toxophore” of the rotenoid structure was reported to be the prenyl-derived ring and the dimethoxy substitution on ring A [6]. So, the rotenoids isolated from BD are noncytotoxic since they lack the isoprenoid residue on ring D or have a monosubstituted or unsubstituted ring A [7]. The chemical marker for BD belongs to rotenoid category; namely, boeravinone B and authentic samples of BD should contain not less than 0.005% boeravinone B [8].

## 3. Status of BD in Traditional Systems of Medicine

As stated earlier, BD is an important herbal constituent of various ayurvedic formulations. It has been used in various formulations meant for inflammation, jaundice, asthma, rheumatism, nephrological disorders, ascites, anemia, and gynecological disorders.  Table 3 enlists various traditional formulations having BD as a main ingredient.

## 4. Ethnopharmacological Reports for BD

Tables 4 and 5 give a detailed account of ethnopharmacological reports for the use of BD throughout India and in other parts of the world, respectively. It can be inferred from the data presented that ethnopharmacological wisdom runs parallel with the modern evidence based system of medicine. Most cited uses were for reproductive system, jaundice, kidney problems, skin troubles, eye diseases, wounds, and inflammation. All of these uses can be verified in the light of current therapeutic studies or the compounds isolated from BD.

## 5. Pharmacological Activities

### 5.1. Immunomodulatory Activity

#### 5.1.1. Immunostimulatory Activity

*In vivo* studies: Mungantiwar and coworkers analyzed the immunomodulation by BD (aqueous extract, 50–200 mg/Kg/day orally) and showed significant leucocytosis and reduced mortality (50%) in pretreated mice using* E. coli-*induced abdominal sepsis stress model. The extract also reversed the elevation in the levels of glucose, cholesterol, SGPT, and BUN and reduction in triglycerides induced by cold and forced swimming stress in rats [9]. The alkaloidal fraction has shown a remarkable effect in leveling the increase in plasma cortisol and averting the decrease in immune system performance in rats [10]. In another study, Sumanth and coworker compared the effect of BD with ashwagandha and found comparable increase in total swimming time in mice when fed with alcoholic extract. The extract showed more potent effect on the count of total WBC, glucose level, and plasma cortisol level. The extract produced macrophage phagocytic activity comparable to the drug levamisole [11]. Mungantiwar and coworkers continued the studies on immunomodulation and found that the alkaloidal fraction (25–100 mg/Kg p.o.) considerably decreased and delayed hypersensitivity reactions in animals. The author recommended that the immunostimulation is due to metabolic alteration of the alkaloid to its active form [12].

BD has been said to possess adaptogenic effects; however the term adaptogen includes a myriad of activities. The term actually infers an overall increase in adaptability of an organism against any type of stress, namely, physical, chemical, or biological. This term can be loosely correlated with the rasayana concept of Ayurveda. Immunomodulation is an important activity of rasayana herbs. Mungantiwar and Sumantha studied the immunomodulation but the activity has been ascribed to crude or semipurified alkaloidal extract. Syringaresinol mono-**β**-D-glucoside (eleutheroside E_1_; acanthoside B), punarnavine, and quercetin are the compounds which have been found to have immunomodulatory activity, isolated from BD or other plants. Apart from quercetin, the other two compounds have been exclusively reported to be present in the roots, the official source of the drug** “**punarnava.**”**

#### 5.1.2. Immunosuppressive Activity

*In vitro* studies: Mehrotra and coworkers studied the immunomodulation produced by an ethanolic extract of BD roots (100 and 500**μ**g/mL) in inhibition of NK cells cytotoxicity, LPS-induced NO production, and quantification of mRNA. The extract prevented* in vitro* cytotoxicity in human NK cells and also inhibited NO generation in mouse macrophage cells along with production of IL-2 and TNF-*α* (MIC ~ 10**μ**g/mL) in human PBMCs. The author suggested good immunosuppressive properties possibly because of alkaloid/lignan [13]. However Pandey and coworkers worked on hexane, chloroform, and ethanol extracts of BD leaves and found inhibition of PHA stimulated proliferation of PBMCs, two-way MLR, NK cell cytotoxicity, and LPS-induced NO production by RAW 264.7 when treated with chloroform and ethanol extracts (5–500**μ**g/mL). Eupalitin-3-*O*-**β**-*D*-galactopyranoside isolated from the ethanolic extract showed more effectiveness. It decreased the production of IL-2 and TNF-*α* in human PBMCs and repressed NF-*κ*B and AP-1, thereby depressing activation and proliferation of T cells. The author suggested specific potential of eupalitin-3-*O*-**β**-*D*-galactopyranoside for immunosuppression [14].

Above reports indicate the immunosuppression by BD; however both reports are from different plant parts. The roots have been the source of two documented immunostimulants, syringaresinol mono-**β**-D glucoside (eleutheroside E1 and acanthoside B) and punarnavine. Furthermore, Pandey and coworkers isolated eupalitin-3-*O*-**β**-*D*-galactopyranoside and ascribed the immunosuppressive property to it [14]. This compound has also been reported to possess antiosteoporotic activity [15]. Osteoporosis is a disorder with an inflammation-aging component and it has been emerged that it has an immune component also. Cytokines which are secreted for immune response are also important for development and activation of osteoclasts besides being critical for the immunity [16]. The immunosuppressive property of eupalitin-3-*O*-**β**-*D*-galactopyranoside could be linked with antiosteoporotic activity shown by BD extract in various cell cultures and* in vitro* studies. BD has been an integral part of traditional and ethnopharmacological medicine for treating rheumatism which is a nonspecific term for medical problems affecting the joints and connective tissues. The evidence for presence of compounds with antiosteoporotic, immunosuppressive, and anti-inflammatory activities approves the use of BD in rheumatic disorders for which it has been known since ancient times.

### 5.2. Anticancer Activity

*In vitro* studies: Srivastava and coworker showed a dose-dependent* in vitro* cytotoxic effect of the extract of the BD root and the leaf in HeLa and U-87 tumor cell lines. Crude ethanolic extract of the root (200**μ**g/mL) and the leaf (300**μ**g/mL) showed 30 and 40% cell death while alkaloidal fraction (300**μ**g/mL) and methotrexate (200 nM) showed 40% cell death [17].

Mehrotra and coworkers analyzed the effect of 95% ethanolic root extract on T cell mitogen PHA, Con-A, and PPD antigen-stimulated proliferation of human PBMC. It inhibited PBMC proliferation induced by all above stimulators and human mixed lymphocyte culture. The extract showed the inhibition of various cell lines (mouse and human) with special mention of lymphoma and leukemic cells [18].

Ahmed-Belkacem and coworkers isolated two rotenoids (boeravinones G and H) from BD roots and found them potential efflux inhibitors for breast cancer resistance protein (ABCG2). The authors also proposed a correlation between structure and activity of compounds having BCRP inhibitory activity [7].

Chopra and coworkers performed bioassay guided fractionation of 95% ethanolic extract of BD root and have observed 30% cell death in HeLa cell line (300**μ**g/mL). Further purification with column chromatography yielded a more potent fraction which has shown 85% and 55% cell death in 72 and 24 h, respectively, at a dose of 300**μ**g/mL [19].

S. Sreeja and coworkers analyzed antiproliferative and antiestrogenic potential of methanolic extract of whole plant of BD in MCF-7 cell line and showed reduction in cell viability (46.8%) in 48 h at 320**μ**g/mL [20]. The extract also showed reduction in estradiol-induced cell proliferation. MCF-7 cells treated with varying concentrations of the extract (20–320**μ**g/mL) showed G_0_-G_1_ arrest by increasing the population of G_0_-G_1_ phase from 69.1% to 75.8%.

*In vivo* studies: Leyon and coworkers studied the effect of aqueous methanolic (3 : 7) extract of BD whole plant on metastasis in a model of B16F10 melanoma in C57BL/6 mice. The extract showed 87% and 95% inhibition of metastasis at 0.5 mg/dose simultaneously and prophylactically. The survival rate of mice was also increased up to 157%. The extract given prophylactically produced 85% reduction in serum parameters indicative of metastasis [21]. Further the author isolated punarnavine from the extract which has shown antibody-dependent cellular and complement mediated cytotoxicity along with enhancement of NK cells activity. Punarnavine increased the production of IL-2 and IFN-*γ* [22]. Levels of GM-CSF and proinflammatory cytokines such as IL-1*α*, IL-6, and TNF-*α* were significantly lowered by punarnavine administration. Further, the author found that prophylactic and simultaneous treatment with punarnavine (40 mg/kg) can restrain the lung melanoma metastasis up to 95.25%–93.9%, respectively, for 10 days after tumor inoculation. Punarnavine administration probably suppresses or downregulates the expression of MMP-2, MMP-9, VEGF, ERK-1, and ERK-2 in the lung tissue of metastasis-induced animals [23].

Manu and coworkers estimated the protection provided by 70% aq. methanolic extract of the whole plant (20 mg/kg, i.p.) in bone marrow and intestine of mice (dosed sublethally by 600 rads in single dose). Total WBC count was reduced by 46.66% in the extract treated group in comparison to 80% in the control group on day 9 after radiation exposure. In the presence of BD extract the effect of radiation on bone marrow cellularity can be seen by only 46% reduction in cellularity compared with 68% reduction in radiation alone. An interesting fact is that, on the 11th day, the count of bone marrow cellularity surpassed the initial value by 9.2%. The elevated level of serum and liver LAP, GPT, and lipid peroxidation after radiation exposure was normalized in the extract treated group [24].

An important indication of BD in traditional medicine is abdominal tumor. Various studies (*in vitro* and* in vivo*) suggest the presence of potential anticancer compounds in various extracts prepared from various plant parts. Manu and coworker isolated the alkaloid punarnavine from the rootsband reported it to have an antimetastatic potential [23]. In another study, boeravinones G and H have shown potential inhibition of drug efflux by breast cancer resistance protein (ABCG2) [7].

Radiotherapy holds an important stake in cancer treatment in spite of the major adverse effect of myelosuppression or immunosuppression which may result in increased susceptibility to infection during the course of cancer treatment. There are several approaches to maintain the immunity level of the cancer patient to improve the overall condition. Herbal formulations containing plant derived immunomodulators might be a considerable approach in this regard. BD offers a multiple target regimen in cancer therapy. It has anticancer, immunomodulatory, and radioprotective activity. So it could be proven to be a beneficial supplement in the cancer therapy.

### 5.3. Antidiabetic and Hypoglycemic Activity

*In vivo* studies: Chude and coworkers showed non-dose-dependent reduction in sugar levels in alloxan induced diabetic rats upon administration of aqueous extract of leaf of BD. They showed 51.95% reduction in sugar level at the 6th hour after administration of 200 mg/Kg extract [25]. In another work, Satheesh and coworkers compared the aqueous extract of the leaves (200 mg/Kg) with glibenclamide (600**μ**g/Kg) in alloxan induced diabetic rats. The extract increases the plasma insulin level from 4.92**μ**U/mL to 10.4**μ**U/mL while glibenclamide attains the peak insulin level of 9.74**μ**U/mL. The extract completely restores initial glucose concentration in 120 min while glibenclamide leaves the level of glucose elevated by almost 10% [26, 27].

BD leaves chloroform extract has shown dose-dependent hypoglycemia in experimentally diabetic rats. Glibenclamide (25**μ**g/Kg) and BD leaf extract (200 mg/Kg) gave the percent glucose reduction of 59.01% and 38.63%, respectively, in the fourth week. The author hypothesized that **β**-cells renewal or some extrapancreatic action is responsible for such activity [28].

*Ex vivo*, Gulati and coworkers have accounted the *α*-glucosidase inhibitory activity for the ethanolic extract (1.72**μ**g/mL) [29].

The author found no traditional or ethnobotanical reports of the antidiabetic activity in BD plant and the formulations containing BD as an ingredient; however the above studies clearly indicate the antihyperglycemic potential of BD. Only one proprietary formulation from Unani-Tibb system (Glucostop) has been indicated in the management of diabetes.

### 5.4. Antifibrinolytic Activity

*In vivo* studies: Srivastava and coworkers studied the effect of BD extract on IUD-induced bleeding in rhesus monkeys and established antifibrinolytic activity of BD extract [30]. Further they evaluated the mechanism of this activity and discovered NAD-dependent-15-hydroxy-prostagtandin dehydrogenase activity in the endometrium [31]. Further exploration showed the role of vascular and t-PA in IUD-fitted menstruating monkeys [32].

Barthwal and Srivastava compared antifibrinolytic agents (*ε*-aminocaproic acid, 100 mg/Kg/day orally, and tranexamic acid, 5.5 mg/Kg/day, i.v.), anti-inflammatory drugs (indomethacin, 1.5 mg/Kg/day, ibuprofen, 3.3 mg/Kg/day, and naproxen, 3 mg/Kg/Day; orally), and root extract of the BD (50 mg/Kg/Day, orally) on various parameters of menstrual cycle in IUD-fitted monkeys. They observed a high increase in duration and loss of iron after IUD insertion. Antifibrinolytic and anti-inflammatory agents reduced the duration and iron loss in menstruation and the activity of t-PA independently whereas root extract of BD (50 mg/Kg, orally) showed greater reduction in the duration of menstrual flow, iron loss, and t-PA activity. The author suggested reduction in t-PA activity leading to decrease in MBL causing reduced MIL in IUD-fitted monkeys [33, 34].

Traditional systems of medicines in India and other parts of the world endorse the use of BD roots in gynecological disorders, for example, abortion, prolapsed uterus, pain in female genital tract, regulation of menstruation, and so forth. Anemia is an easily predictable outcome of most of the gynecological disorders which further compromise the health of females. This set of elaborated studies on the effect of BD extract on menstrual parameters in IUD-fitted monkeys and isolation of phenolic glycoside, punarnavoside, having an antifibrinolytic activity explain its use in diverse gynecological disorders.

### 5.5. Anti-Inflammatory Activity

*In vivo* studies: Mudgal studied the anti-inflammatory effect of aqueous insoluble alcoholic extract of BD in rats. The leaves and flower extracts have shown anti-inflammatory activity by only 55.78% decrease in rat paw edema [35]. Hiruma-Lima and coworkers evaluated BD leaf extracts (juice and lyophilized decoction) for its toxicity and analgesic-anti-inflammatory activities. Juice and lyophilized decoction of the leaves (both 1000 mg/kg; p.o.) produced 50 and 47% inhibition of abdominal writhing in mice in comparison to dipyrone sodium (200 mg/kg). The juice also increased the latency in hot plate test in mice in comparison to morphine. Another important observation was reversal of action of juice by pretreatment with naloxone (5 mg/kg, i.p.), except for the decoction. So the author proposed the opioid related mechanism of antinociception [36]. Asadulla isolated **β**-sitosterol from BD roots and reported 61.29% edema in rats [37].

Inflammation is an important use of BD. This plant is also called sothaghni which means that who alleviate inflammation. Almost all the ayurvedic formulations listed in Table 3 have uses in inflammation. There are several reports of the use of leaves either intact or in a formulation taken orally or applied locally in cases of scorpion and snake bite or for wound healing. Liriodendrin (eleutheroside E; syringaresinol diglucoside), quercetin, and kaempferol have been reported from various extracts from roots and leaves of the plant and have shown potential for anti-inflammatory activity.

### 5.6. Diuretic and Renal Activity

*In vitro* studies: Chauhan and coworkers studied the effect of aqueous extract on growth inhibition of struvite crystals, made up of ammonium magnesium phosphate hexahydrate (AMPH), commonly found in urinary stone (calculi) in women. 0.5 and 1.0% extract administration produced 50 and 71.42% decrease in crystal size. The administration of 1.0% BD extract caused dissolution of crystal by day 4. When studied* in vitro*, 0.5 and 1% extracts have, respectively, shown 88.89 and 138.89% enhanced rate of dissolution in gel at the gel-liquid interface [38].

*In vivo* studies: Mudgal compared the effect of* Convolvulus pluricaulis* and BD against hypotension, potentiation of barbiturate hypnosis, and diuretic and anti-inflammatory activities. The authors found significant diuretic activity in BD root extract (water insoluble portion of alcoholic extract) collected in a rainy season. The authors found 90.3% increase in the volume of urine in rats treated with the extract (300 mg/Kg) whereas extract of leaves and flower showed 67.22% increase in the volume of urine [35].

Singh and coworkers studied the effect of aqueous ethanolic extract on* E. coli*-induced acute pyelonephritis in rats. The extract (50 mg/Kg p.o.) administered twice orally showed 42.85% decrease in number of animals showing signs of renal changes. The administration of the extract (50 mg/Kg p.o.) twice orally showed 99.09% decrease in bacterial count per mL of urine [39].

Wahi and coworkers isolated alkaloid punarnavine and water soluble base choline from BD roots and evaluated them for effects on frogs' heart, frogs' skeletal muscle (rectus abdominis), and diuresis. The authors found significant diuresis after administration of the alkaloid (5 mg/100 g) in rats [40].

Sathyapriya and coworkers evaluated the effect of the aqueous extract of the whole plant of BD on osmotic fragility in erythrocyte from polycystic ESRD patients. It significantly decreased the osmotic fragility in erythrocyte from polycystic ESRD patients. The authors suggested it for a property of altering the erythrocyte membrane composition or a direct/indirect effect on the intracellular sodium and alleviation of oxidative stress [41].

Pareta et al. [42] studied the antioxidant potential of BD extract in urinary stones by means of inhibition of oxidative trauma and kidney cell damage and observed decrease in calcium oxalate deposition.

Yasir et al. [43] reported the ability of ethanolic extract of BD in shrinking crystal size and promoting calcium oxalate dihydrate (COD) crystals formation more than monohydrate (COM) crystals. Singh and coworkers reported the potent renoprotective potentials of BD on alloxan induced diabetic rats indicated by effective glucoregulation, maintenance of serum ionic status and renal Na^+^-K^+^ ATPase activity, and antioxidant status [39].

BD is a well-known diuretic and renoprotective plant in the traditional system of medicine. Studies approving diuretic and kidney stone dissolving properties of BD extracts along with the isolation of a diuretic alkaloid, punarnavine, describe the use of BD in urinary disorders. Formulations containing BD as the main ingredient are routinely used in ascites, anasarca, dropsy, kidney troubles, urinary stones, and swelling of the legs. In case of ascites, cirrhosis is the major cause which is followed by congestive heart failure. Diuretics are the first line of therapy in such cases because all of these diseases involve abnormalities in fluid dynamics in the body; however hepatoprotective activity of BD would be an added benefit in such a case. The use of BD could also be beneficial in congestive heart failure by means of decreasing cardiac load and ACE inhibitor activity.

### 5.7. Hepatoprotective Activity

*In vivo* studies: Gulati and coworkers prepared 50% aqueous ethanolic extract of BD roots and evaluated hepatoprotection at a dose of 100 mg/100 g in hepatotoxicity induced by country made liquor. BD extract reduced the increment in serum parameters indicative of damage to the liver. The increase in SGPT, SAP, triglycerides, and total lipid levels was decreased by almost 50% by administration of BD extract while the level of cholesterol was completely restored. SGOT level was not much affected by BD extract. Histopathological study of the liver showed minimal fatty cysts in BD treated group. The author suggested an additional antilipidemic activity along with hepatoprotective activity [29].

Chandan and coworkers evaluated the 50% aqueous ethanolic extract of BD whole plant given orally for its hepatoprotective activity in carbon tetrachloride induced hepatotoxicity in rats. The extract significantly decreased CCl_4_ induced increase in hexobarbitone sleeping time from 225 min to 200 min. It also lowers the SGPT level from 260**μ**mol/min to 200**μ**mol/min. It showed reduction of the serum levels of SGPT, SGOT, and bilirubin from 270 to 205, 140 to 120, and 1.95 to 1.2**μ**mol, respectively. It also significantly decreased the increase in prothrombin time induced by CCl_4_ from 30.43 to 19.01 sec. In this test, bromosulphalein clearance was reduced to 3 times from 16 times by administration of BD extract. It also almost doubles the flow of bile [44].

Rawat and coworkers studied the effect of various factors for the hepatoprotection by BD extract and found that aqueous extract (2 mL/Kg) of 1–3 cm diameter roots from May displayed significant protection for serum parameters, that is, GOT (82.55%), GPT (74.16%), and ALP (51.47%), but not GLDH and bilirubin in thioacetamide-induced hepatotoxicity. It has been noted in this study that the roots, which were thin, showed maximum protection of serum parameters [45].

Devaki and coworkers studied the effect of ethanolic extract of BD on tissue defense system against ethanol-induced hepatic injury in rats. The administration of BD extract (150 mg/kg/day for 30 days, orally) reversed the increase in the levels of lipid peroxides and increased the activities of superoxide dismutase, catalase, glutathione peroxidase, and glutathione-S-transferase and reduced glutathione levels [46].

Olaleye and coworkers evaluated the aqueous and ethanolic extracts of fresh leaves for antioxidant components and activity by* in vitro* and* in vivo* assays. Antioxidative evaluation of the ethanolic extract has shown appreciable quantities of phenolic and flavonoid content along with vitamins C and E. It also contained selenium and zinc. Pretreatment with BD aqueous and ethanolic extracts reduced enzymatic activities and serum bilirubin caused by acetaminophen. The increase in alkaline phosphatase was reduced by almost 50% by aqueous and ethanolic extracts (both 400 mg/Kg, orally for 7 days) whereas the increase in ALT and AST was decreased by more than 70% and serum LDH level was restored. The increase in TBARS was also neutralized by aqueous and ethanolic extracts [47]. Venkatalakshmi et al. accounted for protection against paracetamol induced hepatotoxicity for BD extracts (Venkatalakshmi, 2011) [48].

Liver is a vital organ of the body and is the first line of defence against xenobiotics. That is why it is targeted by harmful and toxic effects of chemicals. It prepares the body for any onslaught. A good health depends on the health of liver. Jaundice is a disease for which BD has been constantly used either in traditional system of medicine or in ethnopharmacological reports. To prove the efficacy of BD in hepatic disorders and against various hepatotoxins, several workers have evaluated its hepatoprotective potential against different types of toxins. In each and every study the hepatoprotection provided by BD is proved.

### 5.8. Antimicrobial Activity

#### 5.8.1. Antibacterial Activity

*In vitro* studies: the aqueous and ethanolic extracts of BD (whole plant) were found active against* Streptococcus* group (10–19 mm),* Neisseria gonorrhoeae* (ethanolic and water ex.; 5–9 mm),* Salmonella typhimurium* (ethanolic and water ex.; more than 20 mm),* Shigella dysenteriae* (ethanolic and water ex.; more than 20 mm),* Corynebacterium diphtheriae* (water ex.; more than 20 mm), and* Clostridium tetani* (ethanolic ex.; 10–19 mm) [49]. It was observed that ethanolic and aqueous extracts possess antibacterial activity against* Bacillus subtilis* and* Escherichia coli*. The minimum inhibitory concentration of ethanolic extract was found to be 125 and 250**μ**g/mL for* B. subtilis* and* E. coli*, while the aqueous extract showed 250**μ**g/mL for* B. subtilis* and* E. coli,* respectively [50].

Umamaheswari and coworkers studied the effect of various extracts prepared from BD roots against Gram-positive (*Staphylococcus, Bacillus, Streptococcus,* and* Micrococcus*) and Gram-negative (*E. coli, Pseudomonas, Salmonella, Klebsiella, Proteus, Serratia,* and* Shigella*) bacterial strains by observing the zone of inhibition. The ethanol extract of BD leaves demonstrated highest activity [51].

Kant and coworkers established the effectiveness of BD as an adjuvant to chemotherapy in clinical trials conducted on 50 patients newly diagnosed with pulmonary tuberculosis. The clinical recovery rate was faster in BD treated group than in the control. At the end of the 4-week follow-up, 80% of the patients were relieved of cough compared to only 52% in the control group. Similarly, 88% of the patients in the treated group were afebrile in 4 weeks compared to 60% of control. Fever relief was observed in 6 weeks in comparison to 8 weeks in the control groups, respectively. The mean weight gain in the treated group was higher than that in the control group. The rate of sputum conversion was significantly faster in the treated group than in the control group [52].

The rationale for studying the effects on selected microorganisms lies in their potential for causing human diseases. Human pathogenic bacteria cause different types of diseases with varying degree of morbidity and mortality; for example,* Klebsiella* infections have a high mortality rate of approximately 50% even with antimicrobial therapy. The diseases of skin, itching or eczema, conjunctivitis, diarhoea, dysentery, and urinary troubles are caused by the microorganisms.

#### 5.8.2. Antifungal Activity

*In vitro* studies: Agrawal and coworkers evaluated the antifungal activity of ethyl acetate extract of the roots of BD and have shown mycelial growth inhibition for* Microsporum gypseum* (78.83%),* M. fulvum* (62.33%), and* M. canis* (42.30%) in that order at 1 mg/mL. The increase in concentration of extract also inhibited sporulation [53].

*Microsporum gypseum* have been documented as a cause of dermatophytosis which can be characterized by redness of the skin, small papular vesicles, fissures, and scaling. Formulations containing BD (punarnavadyarishta, punarnavadi mandura) have been used for such indications.

### 5.9. Antioxidant Activity

*In vitro* studies: Gacche and Dhole studied the antioxidant and possible anti-inflammatory potentials by evaluating DPPH radical scavenging activity, OH radical scavenging activity, vitamin C content, and total polyphenols. 50% ethanolic extract of BD whole plant showed 0.21 mg/mL IC_50_ for DPPH scavenging, vitamin C content of 22.96 mg/100 g, and 42.5 mg/g polyphenols [54].

Priyadarsini and coworkers have shown dose-dependent radical quenching and reducing power of BD extract against BHA. The IC_50_ of BD extract (49.95 g/mL) was lower than that of BHA (53.27**μ**g/mL) in radical scavenging. The extract has also shown remarkable rise of reducing power as indicated by higher absorbance. Ethanolic extracts of BD also showed potential cytotoxic activity (50**μ**g/mL) against the Vero cell lines [55].

Rachh and coworkers evaluated ethanolic and methanolic extracts of the dried root powder for antioxidant activity. The extract showed good* in vitro* antioxidant activities in terms of ferric reduction and hydrogen peroxide quenching in comparison to ascorbic acid [56].

*In vivo* studies: Satheesh and coworkers demonstrated the antioxidant potential of BD leaf extract in alloxan induced diabetic rats and reported reduction in TBARS and hydroperoxides and considerable enhancement in various enzymes and reduced markers [27].

*In vivo* activity: Vineetha et al. reported the cardioprotective action of BD ethanolic extract against ATO-induced toxicity on various cell organelles in H9c2 cardiomyocytes. The animals demonstrated decreased levels of lactate dehydrogenase, oxidative stress, and calcium influx [57].

Free radicals contain at least one unpaired electron and can exist independently despite of being highly reactive. Free radicals containing oxygen are also called reactive oxygen species (ROS) and have two unpaired electrons. When the free radicals react with a surrounding entity, they generate a new free radical initiating a chain reaction. Inside the physiological system, free radicals are controlled by antioxidant enzymes (superoxide dismutase, catalase, and glutathione peroxidase).

Tumor formation (initiation and promotion) is linked with chromosomal defects and regulation of oncogenes and tumor suppressor genes. It might be probable that endogenous free radicals reactions may cause tumor formation as the case with ionizing radiation.

There is significant connection between fats and oils consumption and death rates from leukaemia and malignant neoplasia which may be due to greater lipid peroxidation [58]. Several studies indicated the role of free radical reactions in arterial wall and serum for diet-derived lipids causing endothelial cell injury [59]. Other diseases having a correlation with oxidative strain are Parkinson's disease, heart failure, myocardial infarction, Alzheimer's disease, and age related symptoms.

BD has been ascribed with various activities which implies the antioxidant activity. The prominent examples are anticancer, hepatoprotective, immunosuppressive, and antidiabetic activities. Moreover it has been characterized as rasayana herb in Ayurveda. All these facts substantiate its use as a rejuvenator and also approve its Sanskrit name “punarnava” which means rejuvenated.

### 5.10. Spasmolytic Activity

*In vivo* studies: Borrelli and coworkers have shown spasmolytic effect of methanolic root extract on exogenous (i.e., acetylcholine, histamine, and barium chloride) as well as electrically stimulated contractions in the isolated ileum. The IC_50_ were 182**μ**g/mL (electric field stimulated), 160**μ**g/mL (acetylcholine-induced), 158**μ**g/mL (histamine-induced), and 168**μ**g/mL (barium chloride-induced). The authors concluded that the spasmolytic activity might involve extracellular calcium, whereas intracellular calcium provides negative modulation for intestinal motility. The authors established that boeravinone E is the most potent spasmolytic compound present in the extract and that nonprenylated rotenoids are the spasmolytic agents in BD root extracts [60].

Involuntary muscle spasm is the symptom of various muscle disorders. Common examples include colic, tremors, neck rigidity/torticollis, pain in female genital tract, threatened miscarriages, palsy/glossal palsy, and paraplegia. Antispasmodics/spasmolytics offer a symptomatic relief in such conditions. The above study demonstrates the effectiveness of BD in spasms caused by different spasmogens and substantiates the use of BD in the above conditions.

### 5.11. Antiasthmatic Activity

*Ex vivo* activity: Irié-N'Guessan et al. documented tracheal relaxation effect against carbachol (1**μ**M) induced trachea contraction [61].

### 5.12. Anticonvulsant Activity

*In vivo* activity: Goel and coworkers have shown anticonvulsant activity in pentylenetetrazol (PTZ) induced seizures in mice and concluded that the calcium antagonist activity is responsible for this since the activity was maintained only by liodendrin-rich fraction, additionally established by anticonvulsant activity in BAY k-8644-induced seizures [62].

## 6. Drug Interactions

BD extracts have several documented activities. To name a few there are diuretic, vasodilatory, immunomodulatory, ACE inhibitory, and anticonvulsant activities. So it can be devised that the products containing BD as main ingredient can have potential for interaction with medications having similar or opposing activities. It is of therapeutic consideration that diuresis, vasodilation, and ACE inhibition are routinely used in cardiac complications and hypertensive crisis and in such cases a delicate balance has to be maintained for patients survival. So it could be advised that the persons with the above kind of cardiac complications should take the formulations containing BD under medical supervision. BD extracts have shown an anticonvulsant activity so it can potentiate the actions of anxiolytics especially benzodiazepines and barbiturates.

In another study quercetin has shown alteration in the bioavailability of tamoxifen upon coadministration. The absolute bioavailability of tamoxifen has been increased from 20% to 60% when administered with 2.5 and 7.5 mg/kg quercetin [63]. Quercetin (10 mg/kg) also increased the bioavailability of simvastatin in pigs when given as a pretreatment by means of increased expression of CYP3A4, the main enzyme responsible for metabolism of simvastatin [64]. Ferreres and coworkers determined the concentration of quercetin in leaves to be up to 150 mg/Kg on dry weight basis [65]. So the formulation containing whole plant or aerial part of BD should be taken with precautions.

## 7. Conclusions

BD is a plant of repute in traditional as well as ethnobotanical systems of medicine in various parts of world. It contains diverse chemical compounds which have shown therapeutic activities, for example, diuresis, anticancer, anti-inflammation, hepatoprotection, and immunomodulation. However, it still has not been able to claim its position in herbal market. In the current scenario of plant based medicinal products, BD can prove to be an effective and affordable commodity for hepatoprotection, diuresis, and immunomodulation. It is also a source of structurally novel rotenoid compounds which can show possibilities to design novel semisynthetic compounds for newer indications.

## Figures and Tables

**Table 1:** Chemical constituents isolated from BD.

Chemical class	Name of compound	Activity reported	Plant part	Reference
Phenolic glycoside	Punarnavoside	Antifibrinolytic	Roots	[66]

C-Methyl flavone	**Borhaavone**	∗	Roots	[67]

Isoflavone	2′-O-Methyl abronisoflavone	∗	∗	[68]

Flavonol	Quercetin, kaempferol	∗	Leaves	[65]

Flavonoid glycoside	3,4-Dihydroxy-5-methoxycinnamoyl rhamnoside	∗	Leaves	[65]
	Quercetin 3-O-rhamnosyl (1→6) galactoside (quercetin 3-O-robinobioside)	∗	Leaves	”
	Eupalitin 3-O-galactosyl (1→2) glucoside	∗		
	Kaempferol 3-O-robinobioside	∗	Leaves	”
	Eupalitin-3-*O*-**β**-*D-*galactopyranoside	∗	Leaves	

Phenolic acid	*trans*-caftaric acid	∗	Roots	”

Rotenoids	Boeravinones A, B, C, D, E, F	∗	Roots	[68–74]
	Boeravinones G, H	Anticancer, spasmolytic	Roots	[68]
	Boeravinones I, J	∗	Roots	[7]
	9-O-Methyl-10-hydroxy coccineone E	∗	Roots	[60]
	Diffusarotenoid	∗	Roots	[75]
	6-O-Demethyl-boeravinone H	∗	Roots	[60]
	10-Demethyl boeravinone C	∗	Roots	”
	Coccineones E, B	∗	Roots	”
	**Boeravinones M, P, Q, R, S**		**Roots**	**[76]**

Xanthone	Boerhavine	∗	Roots	[77]

Lignan	Liriodendrin	Ca^2+^ channel antagonist	Roots	[78]
	Syringaresinol mono-**β**-*D*-glucoside	Ca^2+^ channel antagonist	Roots	”

Purine nucleoside	Hypoxanthine-9-*L*-arabinofuranoside	Cardiotonic	Roots	[79]

Sterol	Boerhavisterol	∗	Roots	[75]

Sterol ester	Boeravilanostenyl benzoate	∗	Roots	”

Ecdysteroid	**β**-Ecdysone	Increases protein synthesis, antidepressant, antistress and immunomodulation, antihyperglycemic, hepatoprotective	Roots	[80, 81]

Fatty acid	Triacont-24-en-1-oic acid	∗	Roots	”

Hydrocarbons	Boeradiffusene	∗	Roots	[75]

**Table 2:** Substitution pattern in rotenoids isolated from BD.

S. No.	Name	R_1_	R_2_	R_3_	R_4_	R_5_	R_6_
Dehydrorotenoid							
1	Boeravinone A	H	H	OCH_3_	H	OH	CH_3_
2	Boeravinone B	H	H	OH	H	OH	CH_3_
3	Boeravinone D	OH	H	OCH_3_	H	OH	CH_3_
4	Boeravinone E	OH	H	OH	H	OH	CH_3_
5	Boeravinone F	OH	H	O	H	OH	CH_3_
6	Boeravinone G	H	OH	OCH_3_	H	OCH_3_	H
7	Boeravinone H	H	OH	OCH_3_	H	OCH_3_	CH_3_
8	Boeravinone I	H	H	OH	OH	OCH_3_	CH_3_
9	9-O-Methyl-10-hydroxy coccineone E	H	H	OH	H	OCH_3_	OH
10	Diffusarotenoid	H	OH	OCOC_4_H_9_	H	OH	CH_3_
11	6-O-Demethyl boeravinone H	H	OH	OH	H	OCH_3_	CH_3_
12	Coccineone B	H	H	OH	H	OH	H
13	Boeravinone M	H	OH	OH	H	OCH_3_	H
14	Boeravinone P	H	H	OCH_3_	H	OH	H
15	Boeravinone Q	H	H	OCH_3_	OCH_3_	OH	CH_3_
16	Boeravinone R	H	H	OH	OCH_3_	OH	CH_3_
17	Boeravinone S	OH	H	OH	H	OH	H

12a-Hydroxy rotenoids							
1	Boeravinone C	H	OH	H	H	OH	CH_3_
2	10-Demethyl boeravinone C	H	OH	H	H	OCH_3_	H
3	Coccineone E	H	H	H	H	OCH_3_	OCH_3_

**Table 3:** Ayurvedic formulations containing BD as main ingredient.

S. No.	Name of formulation	Uses	Reference
Ayurvedic formulations			
1	Punarnavadyarishta	Heart disease, anaemia, inflammation, splenomegaly, vertigo, hard stools, chronic obstructive jaundice/chlorosis/advanced stage of jaundice, abdominal lump, fistula-in-ano, cough, dyspnoea/asthma, malabsorption syndrome, diseases of skin, and itching	(Bhaisajyaratnavali, Sotharogadhikara: 192–196)
2	Punarnava guggulu	Gout, inguinoscrotal swellings, sciatica, pain in calves-thighs-back-sacral and bladder region, and rheumatism	(Bharat Bhaishajya Ratnakar, Trtiya bhaga: 4012)
3	Punarnavasava	Dyspepsia, abdominal lump, diseases of abdomen/enlargement of abdomen, inflammation, disorder of spleen and liver, and all types of disorders with difficult prognosis	(Bhaisajyaratnavali, Sotharogadhikara: 197–201)
4	Punarnavadi kvatha curna	Generalized tremors, ascites, cough, colicky pain, dyspnea/asthma, and anaemia	(Bhaisajyaratnavali, Udararogadhikara: 43-44)
5	Punarnavastaka kvatha curna	Ascites, anasarca, cough, dyspnea/asthma, and colicky pain	(Chakradatta, Sothacikitsa: 10)
6	Punarnavadi mandura	Anaemia, malabsorption syndrome, inflammation, splenic disease, intermittent fever, haemorrhoids, diseases of skin, and helminthiasis/worm infestation	(Carakasamhit, Cikitsasthana, Adhyaya 16: 93–95)
7	Sukumara ghrita	Constipation, diseases of abdomen/enlargement of abdomen, abdominal lump, splenic disease, abscess, edema, pain in female genital tract, haemorrhoids, inguinoscrotal swellings, diseases due to vata dosha, and gout	(Sahasrayoga, Ghrtaprakarana: 4)
6	Maha Narayan Taila	Facial palsy, deafness, paraplegia, tremors, neck rigidity/torticollis, lock jaw, wasting of one limb, oligospermia, infertility, headache, glossal palsy, dental pain, mania/psychosis, hump-back/kyphosis, fever, senility/progeriasis, emaciation, tendon tear, and bone fracture	(Bhaisajyaratnavali, Vatavyadhyadhikara: 151–162)
9	Sothaghna Lepa	All types of inflammation	(Sarngadharasamhita, Uttarakhanda, Adhyaya 11: 3)
10	Varuni	Rhinitis and pain	[82]

Siddha formulation			
1	Talakacenturam	Diseases due to heat/pitta humour, wheezing, jaundice, arthritis/arthralgia, itching, oliguria/anuria, ascites and diseases due to vāta humour	(Anonymous, 2008)

**Table 4:** Ethnopharmacological reports of uses of BD in various parts of India.

S. No.	Disease/organ involved	Plant part/formulation/dose	Method	Geographical area/location	Reference
1	Male reproductive system	Root/15 g powder with 100 mL cow milk twice daily 20 g powder with 250 mL cow milk twice daily 20 g powder: 15 mL honey with 250 mL cow milk, twice daily	Interview with Vaidyas	Uttar Pradesh (5 Districts)	[83]

2	Female reproductive system	Root/decoction	Interview with elderly women	Uttar Pradesh	[84]
		Root/2 g paste with cow's milk	Questionnaire, survey with traditional practitioners	Churu district; Rajasthan	[85]
		Plant/powder, twice a day for one month	Discussion with elderly women and tribal practitioners	Warli tribe of Maharashtra	[86]
		Root/paste	Survey among aborigine peoples	Tribals of Maharashtra	[87]

3	Hepatic system/Jaundice	Root/decoction	Field surveys	Tribe of Dehradun	[88]
		Leaves/∗	Field surveys	Tribe of Madhya Pradesh	[89]
		Root/decoction	Ethnobotanical survey with local Vaidya	Rewa, Madhya Pradesh	[90]
		Whole plant/∗	Tribal physician	Tribes of Central India	[91]
		Leaves/extract	Interview with men and women between 20 and 80 years	Tribes of Kerala	[92]
		Root/decoction	Field survey	Kanyakumari, Tamil Nadu	[93]
		Root/∗	Questionnaire, interviews, and discussions with tribes	Tamil Nadu	[94]
		∗/∗	Field trips and local villagers	Assam	[95]
		Whole plant/infusion; orally, on empty stomach	Interviews with the traditional practitioners	Assam	[96]
		Leaves/juice, orally twice daily	Enlisting plants	Tribes of Meghalaya	[97]

4	Diuretic/ nephrological system	Root/decoction, daily for one month	Survey with local healers and herbalists, priests, hakims, and Vaidyas	Muzaffarnagar district, Uttar Pradesh	[98]
		Leaves/∗	Field survey	Tribes of Madhya Pradesh	[89]
		Whole plant/∗	Tribal physicians	Tribes of Central India	[91]
		Root/∗	Interviews with local villagers and herbalists	Tribes of Maharashtra	Petkar, 2002
		Whole plant/decoction	Field trips and interview with the tribal	Tribes of Northeast Gujarat	Bhat, 2002
		Root or tender shoots/decoction, One teaspoonful twice daily	10-year ethnobotanical field survey	Tribes of Tamil Nadu	[99]
		Leaves/decoction	Interviews with local Vaidyas	Tamil Nadu	[100]
		Whole plant/∗	Interviews with local people, Vaidyas	Karnataka	[101]

5	Wound healing	Root/∗	Interviews with women having knowledge of medicinal plants	Garhwal Himalaya, Uttaranchal	[102]
		Leaves/paste	Interviews with tribal medicine men	Tribes of Madhya Pradesh	[103]
		Leaves/paste	Field surveys	Tribes of Tamil Nadu	[104]

6	Respiratory system	Plant/infusion	Field surveys	Tribes of Dehradun	[88]
		Root/∗	∗	Chhattisgarh	[105]
		Whole plant/decoction (15–20 mL) twice a day	Field trips and interview with the tribal	Maharashtra	[106]
		Root/powder with equal amount of sugar candy	Questionnaire and surveys with traditional healers and field survey	Orissa	[107]
		Whole plant/∗	Interviews with local people, Vaidyas	Karnataka	[101]
		Root/juice mixed with crushed chillies, taken orally twice daily	Enlisting plants	Tribes of Meghalaya	[97]
		Root/juice, 15 mL taken twice a day orally	Interviews with tribal and nontribal inhabitants and with herbalists	Tribes of Andhra Pradesh	[108]

7	Insect/scorpion/ snake bite	Leaves/whole leaves chewed	5-year survey with medicine men, priests	Rajsthan	[109]
		Leaves/∗	Ethnobotanical survey with local Vaidyas	Rewa, Madhya Pradesh	[90]
		Plant/paste with black pepper, taken orally and applied locally	Questionnaire survey with traditional healers	Orissa	[107]
		Leaves/juice; 2-3 times applied locally and taken orally for 7 days	Interview with traditional healer	Assam	[110]

8	Ophthalmia	Leaves/extract	Field surveys	Tribes of Dehradun	[88]
		Root and leaves/ash	Questionnaire survey with rural folk	Rajsthan	[85]
		Root/decoction	Ethnobotanical survey with local Vaidyas	Rewa, Madhya Pradesh	[90]
		Root/∗	∗	Chhattisgarh	[105]
		Root/paste	Ethnomedicinal survey among aborigine peoples	Maharashtra	[87]
		Leaves/juice	Participatory rural appraisal and questionnaire survey	Kerala	[111]
		Leaves/juice with cow milk is applied on eyelids	Questionnaire survey with traditional healers	Orissa	[107]

9	Skin disorders	Leaves/powder with mustard oil	Questionnaire survey with experienced traditional practitioners	Rajsthan	[85]
		Root bark/paste	Elderly persons	Tamil Nadu	[112]
		Whole plant/∗	Tribal physician	Tribals of Central India	[91]

10	Rheumatism	Leaves boiled with rice, garlic, and water are rubbed on the body	Questionnaire survey with experienced traditional practitioners	Rajsthan	[85]
		Root/decoction	Field survey	Kanyakumari	[93]
		Leaves/decoction	Interviews with local Vaidyas	Tamil Nadu	[100]

11	Cardiovascular system	Whole plant/∗	Interviews with local people, Vaidyas	Karnataka	[101]
		Leaves/∗	Interviews using unstructured questionnaire	Kerala/Western Ghats	[113]
		Leaves/cooked as a curry	Enlisting plants	Tribals of Meghalaya	[97]

12	Inflammation/ edema/ arthritis	Root/decoction	Ethnomedicinal survey among aborigine peoples	Tribes of Maharashtra	[87]
		Root decoction (along with other herbs)	Questionnaires among the tribal practitioners	Tribes of Tamil Nadu	[114]
		Leaves/juice with black pepper, taken orally on empty stomach, twice a day for 7 days	Questionnaire, survey with traditional healers	Orissa	[107]
		Leaves/juice; taken orally 2-3 times and applied locally	Interview with traditional healer	Assam	[110]
		Leaves/juice, thrice a day	Field survey	Uttaranchal	[115]

**Table 5:** Ethnopharmacological reports of uses of BD in various parts of the world.

S. No.	Location	Plant part and formulation	Uses	Reference
1	Northeastern Brazil	Sitting baths made with tea from the bark	Problems of genitourinary system in females and inflammations	[116]

2	Nigeria	Whole plant	Treatment of threatened miscarriage	[117]

3	Tropical Africa	Boiled roots	Ulcers, abscesses, and Guinea worm disease	[118]
		Boiled roots and leaves	Expectorant and febrifuge and, in large doses, emetic	”
		Decoction of aerial parts	Gastrointestinal pains, convulsions, intestinal worms, and regulation of menstruation	”

4	Ghana	Root decoction	Anaemia, heart troubles, palpitations, and jaundice and applied externally to yaws	[118]
		Powdered root with butter or oil	Abdominal tumours	”

5	Congo	Root sap is rubbed on the neck and throat in water or palm oil or in a decoction	Mumps, laryngitis, and burns, spleen troubles, diarrhoea, dysentery, haematuria, and gonorrhea	[118]

6	Democratic Republic of Congo	Leaf decoction	Gonorrhoea and pain	[118]

7	Angola	Root decoction	Jaundice	[118]

8	Berg Damara people, Namibia	Chew or boil the root	Gastroenteritic problems	[118]
	Damara people, Namibia	Tea made from the root	Prolapsed uterus	”

9	Buner District, NWFP, Pakistan	Bandage of roots crushed in boiled milk used externally	Ulcers/interviews with the local inhabitants, selected informants, the herbalists “Hakims,” and sellers” pansaris'	[119]

10	Chakma, Arma, and Tripura community, Chittagong Hill tracts, Bangladesh	Whole plant, juice, and powder	Blood purification, urinary troubles, contraception, and jaundice/questionnaire survey with local herbalists	[120]
	”	Whole plant	Pulmonary tuberculosis	”
	”	Plant powder	Abdominal tumor, dysentery, and renal diseases	”
	”	Flowers and seeds	Contraceptives	”
	”	Roots	Jaundice, anemia, gonorrhea, blood purification, and as stimulant	”
